# Resolutions Declaring the Rate of Compensation for Examining Applicants for Insurance

**Published:** 1869-02

**Authors:** H. O. Walker, Carl Brumme

**Affiliations:** Sec’y.; Vice President


### Resolutions Declaring the Rate of Compensation for Examining Applicants for Insurance.

At a meeting of the Wayne County Medical Society, held in Detroit, on Wednesday, January 6, 1869, the following report of a committee appointed to fix a rate of compensation for examining applicants for life insurance, was adopted:

*Mr. President and Gentlemen of the Wayne County Medical Society:*

Your committee, to whom was referred for report the question, "What should constitute the proper medical charges for examination of applicants for life insurance, and what should constitute the proper fee for attestation as medical attendant," respectfully submit the following:

The tables of longevity of man are based on the initial of health, and from this stand point all calculations must arise. To obtain this initial of health, the life insurance companies have always, and must necessarily rely upon the opinions of the medical profession, who are the only authority. In other words, the medical profession are the guardians of the whole science of successful life insurance.

Occupying, therefore, as they do, this prominent position, it should follow that in the emoluments arising therefrom, the examining physician should be properly and liberally remunerated for his services. Is he so?

Life assurance companies are now paying for examinations, which inform them of the standard of health of the person they propose to insure, the sum of from two to three dollars. No difference is made whether the risk be for one hundred dollars or twenty thousand. The agent may make his three to five hundred dollars on the amount insured. The officers have high salaries, and the stockholders large and frequent dividends, but the miserable pittance paid the physician is considered a large fee. The profession have themselves to blame only for this state of affairs, and to remedy this is the object of this report. A united effort is only necessary.

Your committee are of the opinion that now is the time, not only for the medical profession of the city, but for the profession of the whole State, (and we might say the whole United States,) to demand that remuneration which would be an equivalent for the service rendered.

What sum should, then, constitute a proper remunerative fee? Upon a careful examination of the whole question of fees, a sum of not less than *four dollars* should be charged for each and every primary examination.

Were the life insurance companies of this city all to combine, and employ only one examiner, at a fee of three dollars for each examination, his income would even then be less than that of any physician of moderate practice, and not entirely satisfactory for an annual professional income, considering the value of the talent employed.

In certifying to the health of the applicant for life insurance by his usual medical attendant without fee, except as obtained from the applicant, a great hardship has been thrown on the profession, without the least consideration of the relation of medical adviser and patient. We are, with surprising coolness, asked to betray that which has been confidentially placed in our charge, and so deprive our patrons of the advantages of life assurance, or to falsify our statements to the interrogatories put, in order that he may be insured, and even then are told the company does not pay, but the applicant should. He does it with pleasure, if insured, but is indignant at the charges if rejected by his physician's statement, and, of course, refuses the bill.

How, then, are the profession to remedy and protect themselves against this imposition?

1st. By refusing to give any such certificate unless with the verbal or written assent of the person to be insured.

2d. By refusing such certificate until the payment of a fee of not less than *three dollars*, if it can be given in the office of the attending physician, and if a visit to the residence of the applicant be necessary, then a further fee of *two dollars* be added, and the whole to be paid by the assurance company.

In making these demands, the medical profession require nothing but what is just, and what every life insurance company can easily and readily pay. They ask nothing more than a united effort will necessarily receive, and speedily accomplish. We therefore recommend the adoption of the following resolutions:

*Resolved*, That the fee for examination for life insurance companies shall be the sum of *four dollars* for each and every primary examination.

*Resolved*, That we will not give a certificate as "family physician" without the verbal or written assent of the person to be insured, and even then reserving the right to withhold the same if for the interest of the family of the applicant.

*Resolved*, That the fee for such certificate shall not be less than *three dollars*, if the blanks can be filled in the physician's office; and if further labor be entailed, a further fee of *two dollars* be added, the whole to be paid by the insurance company; and

*Whereas*, A united action of the profession is necessary to perfect the above recommendation, and carry the resolutions into proper effect; therefore,

*Resolved*, That the Wayne County Medical Society respectfully and earnestly ask of the profession, not members of the Society, in the city of Detroit, to cordially unite for the common good in our effort for remunerative fees for examinations and certificates in life insurance, as we believe the charges demanded are equable and just.

*Resolved*, That the physicians of the State be requested to take like action in this matter; and that to further the same, your committee be empowered to present this report and resolutions to the State Medical Society at its next meeting in June.

*Resolved*, That the time for the taking of effect of the resolutions regulating the rate of fees be the first day of February, 1869, and until that time no member of this Society will be expected to contract, for any length of time, with any company, at a less rate than the tariff fee.

*Resolved*, That the Secretary send a copy of this report and resolutions to every medical journal in the United States, to the public newspapers of this city, and a copy of the resolutions fixing the tariff of fees and time of taking effect to each life insurance agent in the city, and the home offices, and that the Treasurer be authorized to pay all expenses attendant thereon.

WILLIAM BRODIE,<br>J. F. NOYES,<br>H. F. LYSTER, } *Committee.*

H. O. WALKER, *Sec'y.*

CARL BRUMME, *Vice President.*

